# Combination of Two but Not Three Current Targeted Drugs Can Improve Therapy of Chronic Myeloid Leukemia

**DOI:** 10.1371/journal.pone.0004423

**Published:** 2009-02-10

**Authors:** Natalia L. Komarova, Allen A. Katouli, Dominik Wodarz

**Affiliations:** 1 Department of Mathematics, University of California Irvine, Irvine, California, United States of America; 2 Department of Ecology and Evolutionary Biology, University of California Irvine, Irvine, California, United States of America; University of Cape Town, South Africa

## Abstract

Chronic myeloid leukemia (CML) is a cancer of the hematopoietic system and has been treated with the drug Imatinib relatively successfully. Drug resistance, acquired by mutations, is an obstacle to success. Two additional drugs are now considered and could be combined with Imatinib to prevent resistance, Dasatinib and Nilotinib. While most mutations conferring resistance to one drug do not confer resistance to the other drugs, there is one mutation (T315I) that induces resistance against all three drugs. Using computational methods, the combination of two drugs is found to increase the probability of treatment success despite this cross-resistance. Combining more than two drugs, however, does not provide further advantages. We also explore possible combination therapies using drugs currently under development. We conclude that among the targeted drugs currently available for the treamtent of CML, only the two most effective ones should be used in combination for the prevention of drug resistance.

## Introduction

Chronic myelogenous leukemia (CML) is a cancer of the hematopoietic system and is initiated and driven by the product of the BCR-ABL fusion gene [1]–[4]. It proceeds in three stages: the chronic phase in which the number of cells is relatively low and the degree of cellular differentiation is relatively high; the accelerated phase during which the number of cells starts to rise to higher levels and the degree of differentiation declines; and blast crisis, which is characterized by explosive cell growth and a low degree of differentiation. Small molecules that specifically target the BCR-ABL gene product provide a successful treatment approach which can lead to a reduction of BCR-ABL+ cells below detectable levels, at least during the early stages of the disease. The drug Imatinib has been mostly used in this respect [4]–[9]. As the disease advances, however, the chances of treatment failure rise due to the presence of drug resistant mutants that are generated mostly through point mutations, but also through gene duplications [5], [6], [8]–[15]. Drug resistance can potentially be overcome by the combination of multiple drugs, where a mutation that confers resistance against one drug does not confer resistance against any of the other drugs in use. This is applied successfully in HIV infection [16]. Computational work has shown that a combination of three or four different drugs against CML could similarly overcome the resistance problem, even during advanced stages [17]. In addition to Imatinib, the drugs Dasatinib and Nilotinib are alternative inhibitors of the BCR-ABL gene product [18]–[21]. The reality, however, is that one mutation (T315I) can confer resistance against all those drugs [18]–[21]. We refer to this as cross resistance. In addition to this one mutation, more than 50 mutations have been identified that confer resistance against one of the drugs (in particular imatinib), but not against the others [20], [22]. Given this situation, the question arises whether a combination of those three drugs can improve treatment outcome. Here we examine this question with a mathematical model.

## Results and Discussion

We construct and analyze a mathematical model that is based on a previously published computational framework [17]. This is a stochastic, continuous time, birth-death process with mutations, by which we describe the dynamics of a heterogeneous population of cancer cells. The cells can divide, die, and mutate with given probabilities. In particular, upon each division, a cell has a probability to acquire resistance against a given drug by mutation. Before the start of treatment, the colony undergoes a clonal expansion until it reaches size *N.* At this time, therapy is started; the drug(s) are assumed to increase the death rate of susceptible cells. This could potentially lead to a significant reduction of BCR-ABL+ cells in the absence of resistance. We calculate the probability that drug resistant mutants will lead to treatment failure, and the complementary probability of treatment success.

The model includes several biological parameters. The colony size at start of treatment is assumed to vary within 10^8^–10^13^ cells. The death rate of cells in the absence of treatment is between 0 and 70% of their birth rate. In the presence of drug therapy, susceptible cells have an additional, drug-induced death rate which can be one to ten times greater than the cells' division rate. In the presence of multiple drugs, the susceptible cells are killed at the rate given by the maximum drug-induced rate in the combination. We assume that there are of the order 10–100 different point mutations that can confer resistance against only one of the drugs. There is also one particular mutation which can confer resistance against all drugs in use. Our general modeling approach continues the tradition of Goldie and Coldman [23]–[25] in that we view a cancerous colony as a stochastic birth-death process; the main differences include the possibility to use drugs in combination rather than sequentially; the existence of combinatorial mutation networks and a non-zero cellular death rate. More mathematical details of the model are provided in the supporting online Material S1.

**Figure 1:**
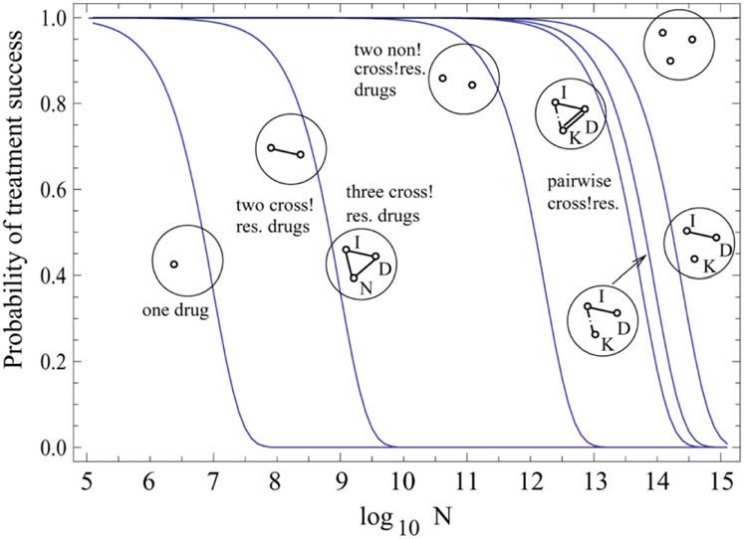
The probability of treatment success is plotted as a function of the colony size, *N*. Different curves correspond to different combination treatments, with one, two and three drugs. The cross-resistance networks are presented by using connected and disconnected nodes. The number of nodes corresponds to the number of drugs used. Connected nodes correspond to the existence of a cross-resistant mutation. Identical connecting lines indicate that the same mutation confers cross-resistance to all connected drugs. Different (single, double, dashed) lines correspond to different mutations. Simulation parameters are as follows: μ = 10^−9^, *k = 100*, *M_0_ = 100*, *d/l = 0.5*, *h/l = 3*. The symbols “I”, “D”, “N”, and “K” stand for “Imatinib”, “Dasatinib”, “Nilotinib” and a future drug which can bind to T315 mutants.

**Figure 2:**
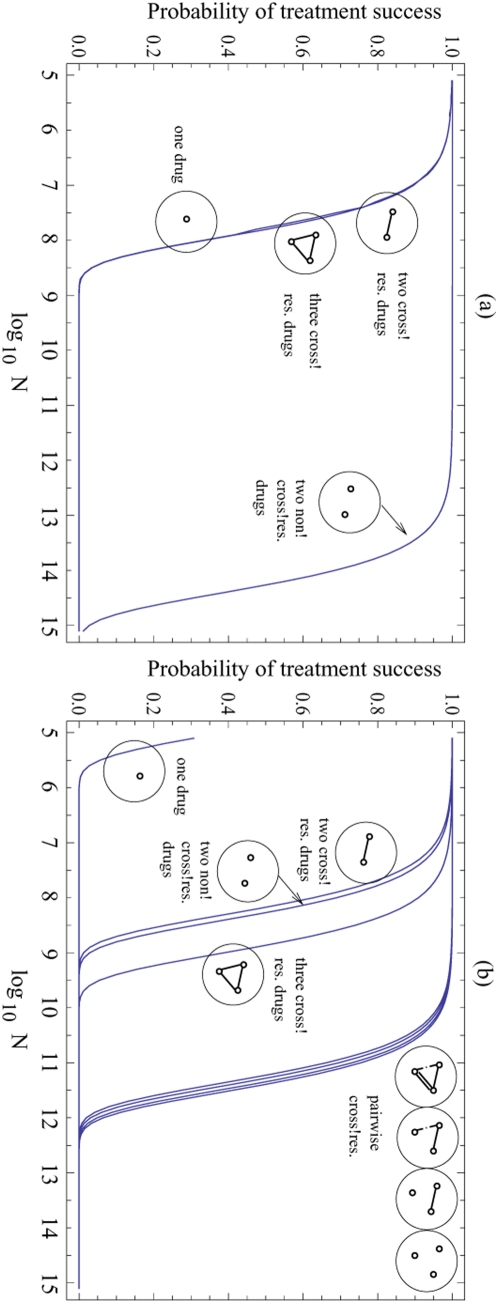
The probability of treatment success is plotted as a function of the colony size, *N*. (a) The number of non-cross-resistant mutations is low (*k = 10*) and the mutation rate for cross-resistance is 10 times higher than in figure 1. Conclusion: combining more than one cross-resistant drugs does not improve the chances of treatment success. (b) The number of non-cross-resistant mutations is high, *k = 10^4^*. Conclusion: combining three cross-resistant drugs improves the chances of treatment success compared with two cross-resistant or non-cross-resistant drugs (which in turn is better than using only one drug). The rest of parameters are as in figure 1.

First, consider the combination of two drugs. For reference, Figure 1 shows the probability of treatment success for one and two drugs assuming the absence of cross resistance. This is compared to the cross-resistance scenario. While the probability of treatment success is lower in the presence than in the absence of cross resistance, combining two drugs with cross-resistance clearly improves the probability of treatment success relative to the use of only one drug. The reason is that it is much more likely to acquire a mutation that confers resistance against only one drug than to acquire the cross-resistance mutation. This is because only one specific mutation can lead to cross-resistance, while many mutations can confer resistance against only one drug. Hence, for most mutations, combination therapy will not be challenged by cross-resistance. On a qualitative level, this result does not depend on the kinetic parameters of the model, such as the division and death rates. The advantage of combining two drugs becomes insignificant if the number of mutations that confer resistance to only one drug is very low (Figure 2b), or if the rate at which the cross-resistance mutation is acquired is relatively high. If the number of mutations that confer resistance against only one drug is on the order of 50–100, and if the rate at which resistance mutations are generated is on the order of 10^−6^–10^−9^, however, the model suggests that combining two drugs is advantageous to the patient, even if cross-resistance is possible.

**Figure 3:**
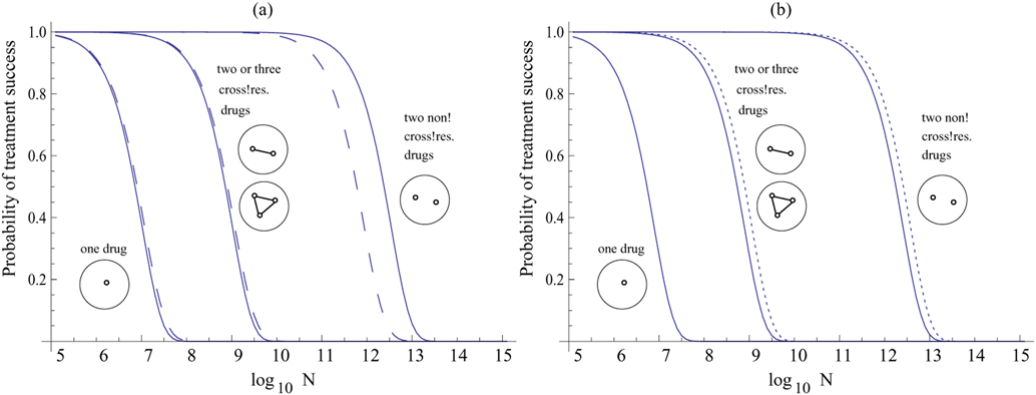
The dependence of treatment success on parameters. (a) The role of the natural death-rate of the cancer cells: *d/l = 0* (solid lines) and *d/l = 0.8* (dashed lines); *h/l = 10*. (b) The role of the drug-induced death rate: *h/l = 3* (solid lines) and *h/l = 400* (dotted lines); *d/l = 0*. Other parameters are the same as in figure 1.

Next, consider the combination of three drugs, i.e. Imatinib, Dasatinib and Nilotinib. The model shows that combining three drugs will not lead to any further advantage compared to the combination of two drugs (Figure 1). For triple combination therapy to be advantageous, most resistant cells must harbor mutations that render them resistant against two of the drugs (but not the third one). Accumulating two separate resistance mutations, however, is a relatively rare event. It is much more likely that a cell acquires the single cross-resistance mutation. Hence, triple combination therapy does not improve the probability of treatment success compared to double combination therapy. Triple combination therapy can only provide an additional advantage if the number of mutations that confer resistance against only one drug is unrealistically high (Figure 2b). Assuming reasonable values for the cellular division, death, and mutation rates, there must be at least *k = 1000* mutations that confer resistance against only one drug for triple combination therapy to somewhat improve the chances of treatment success. The improvement becomes significant for *k* = 10,000 or more mutations (Figure 2b). For such high values of *k* we observe that the effect of cross-resistance is insignificant and treatment failure occurs as a result of mutations that confer resistance to one drug at a time. In this case combining three (cross-resistant) drugs gives a better outcome than two non-cross-resistant drugs (see figure 2b). Again, these results do not qualitatively depend on the kinetic parameters of the model. Figure 3 demonstrates how the probability of treatment success for one, two and three cross-resistant drug therapies changes as a function of the natural cancer cell death rate (a) and drug-induced death rate (b).

In summary, the analysis shows that in the context of the currently available drugs for CML therapy (Imatinib, Dasatinib, and Nilotinib), combining two of them can provide an advantage over using one drug alone. Recent experimental data support this notion [19]. However, combining all of these drugs does not improve the chances of treatment success beyond double combination therapy. Hence, the two most effective drugs should be given simultaneously to treat CML.

While these results are robust and do not break down in the context of biologically reasonable values of the cell death rates and drug-induced death rates (figure 3), the largest degree of uncertainty exists regarding the relationship between the number of tumor cells and the probability of treatment success. As evident in Figure 1, the probability of treatment success is only high if the number of tumor cells upon start of therapy is no larger than 10^10^. However, many more tumor cells than this are found in patients when the disease becomes detectable, and especially during more advanced stages. On the other hand, the tumor cell population is heterogeneous, and it is not clear to what extent different cell populations contribute to the disease dynamics [26], [27]. For example, if only the more primitive tumor stem cells and progenitor cells are important determinants of tumor growth, then the effective population size that our model refers to is significantly lower than the overall number of tumor cells detected in a patient.

In general, we examined all possible resistance networks for three-drug therapies. These networks are represented by circles in Figures 1 & 2. They consist of nodes corresponding to different drugs; each pair of circles is connected if there exists a mutation event that confers cross-resistance against the two drugs. Different lines (single, double, dashed) corespond to different mutations. There are five possible three-drug cross-resistance networks, including the absence of resistance and triple cross-resistance, exemplified by the case of Imatinib, Dasatinib and Nilotinib. Here we examine all intermediate possibilities. Let us suppose that there is some cross-resistance between drug K and, say, I. In other words, even though the T315I mutation does not confer resistance to K, there may be a different mutation which makes the cell resistant to both K and I, but not to D. In this situation, treating with I and K is obviously better than just treating with I (this follows from our previous results), but what is interesting, adding D does give an advantage in this case. Adding D will even give an advantage if there is a third mutation which confers resistance to both K and D (but not I). In other words, even though treating with three drugs characterized by triple cross-resistance does not improve the chances of treatment success compared to two such drugs, treating with three *pairwise*-cross-resistant drugs is advantageous compared to treating with two such drugs.

General cross-resistance networks have relevance for future generation treatment options. If drugs are used that show no cross resistance with Imatinib, Dasatinib, and Nilotinib, such as VX-680 [22], [28]–[33], would it be advantageous to combine such a drug with two or more drugs that do not inhibit the T315I mutation? Our calculations show that it is advantageous to combine such a future generation drug (call it drug “K”) with two drugs that cannot act on the T315I mutation , say, Imatinib (I) and Dasatinib (D). This is seen for example in figure 1 when we compare the probability of treatment success for two cross-resistant drugs with that for “pairwise cross-resistant” drugs. We observe that any of the pairwise cross-resistant combinations of three drugs gives a significant improvement. Note that adding a third cross-resistant drug does not improve the chances of successful therapy. In future work, we will explore the possibility of treatment combinations of currently used drugs, as well drugs under development, based on in vitro data such as published in [19], [34].

To conclude, we performed quantitative studies of drug cross-resistance in CML treatment. The mathematical framework created here is general enough to be applied to other cancers. However, the model analyzed is specific to CML and the existing drugs Imatinib, Nilotinib and Dasatinib. We predict that (i) combining two cross-resistant drugs improves the chances of treatment success, (ii) combining more than two drugs that do not inhibit the T315I mutation does not increase the probability of treatment success compared to combinations of two drugs, and (iii) once a drug effective against T315I mutants becomes available, the most effective treatment strategy is to combine that drug with two of the three presently existing drugs.

## Materials and Methods

Our work is based on mathematical analysis of stochastic processes, describing the dynamics of drug sensitive and drug resistant cell populations, both in the pre-treatment phase and during therapy. Mathematical details are given in the supporting online Material S1.

## Supporting Information

Material S1 Supporting online material
(0.08 MB PDF)
