# Supplementary material for: Combination of Two but Not Three Current Targeted Drugs Can Improve Therapy of Chronic Myeloid Leukemia
Source: PLoS One. 2009 Feb 10;4(2):e4423. doi: 10.1371/journal.pone.0004423 (PMC2635955; doi:10.1371/journal.pone.0004423)
Supplement: Material S1 — Supporting online material (0.08 MB PDF) [file pone.0004423.s001.pdf]

Combination of two but not three current targeted drugs can improve therapy of chronic myeloid leukemia

Natalia L. Komarova, Allen A. Katouli, and Dominik Wodarz

Supporting online materials

Combinatorial mutation diagrams and the formulation of the stochastic process. Following the method developed in [1], [2], we separate all cells in $2^m + 1$ resistance classes, where m is the number of drugs used. The classes are numbered by binary strings of length m, see figure 1; for example, the all zeros string corresponds to the fully susceptible type, and the all ones string - to the fully resistant type. We denote by i_s the number of cells of resistance class s, and let $\varphi_{i_0,j_0,\ldots,i_n,j_n}(t)$ be the probability that at time t there are i_s cells of resistance class s. We define the following probability generating function:

$$\Psi(\xi_0,\ldots,\xi_n;t) = \sum_{i_0,i_1,\ldots,i_n} \varphi_{i_0,\ldots,i_n}(t)\xi_0^{i_0}\ldots\xi_n^{i_n}.$$

Suppose that cancerous cells divide with rate l_s and die with rate D_s. These coefficients can depend on the treatment dose; in particular, the death rate of cells is comprised of the "natural" rate of cell death in an untreated tumor and the action of the drug(s), if any, upon the cells. The mutation rates are defined for all the edges of diagram of figure 1, by $u_{i\to j}$, where the subscript for each mutation rate u represents the binary indices of the starting and the end point of the mutation process. Figure 1 presents all possible mutations types; in reality some of them may of course have zero rates. The Kolmogorov forward equation for the processes of growth, death and mutation are as follows:

$$\frac{\partial\Psi}{\partial t} = \sum_{s=0}^{2^m} \frac{\partial\Psi}{\partial\xi_s}\left(l_s(1-\sum_j u^{out}_{s\to j})\xi_s^2 + \sum_j l_s u^{out}_{s\to j}\xi_j^{out} + D_s - (l_s+D_s)\xi_s \right);$$

here, $u^{out}_{s\to j}$ denotes the mutation rates for all arrows originating at s. Note that here we do not consider the processes of quiescence and awakening of cells (see [4], [3]). While important for the timing of treatment, these processes do not contribute to the overall probability of treatment success. Therefore, for the purposes of this study, they are omitted. Following the standard technique for transport-type equations, we have the following system of equations for characteristics:

$$\dot{\xi}_s = l_s(1-\sum_j u^{out}_{s\to j})\xi_s^2 + \left(\sum_j l_s u^{out}_{s\to j}\xi_j^{out} - (l_s+D_s)\right)\xi_s + D_s.$$

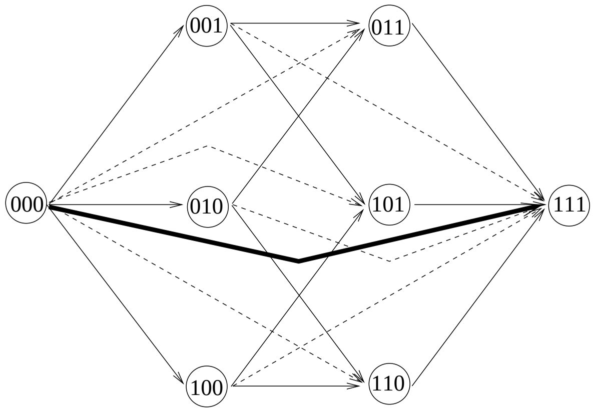

Figure 1: Combinatorial mutation diagram for different resistance classes. Treatment with $m = 3$ drugs. One-drug mutations are denoted by thin solid arrows, two-drug mutations by dashed arrows, and three-drug mutations by a thick solid arrow.

If initially there are M_0 cells of resistance type 0, then we have

$$\Psi(\ldots;0) = \xi_0^{M_0},$$

that is, to find the function $\Psi(\bar{\xi}_0, \ldots, \bar{\xi}_n; \bar{t})$, we need to solve the characteristics equations with the initial conditions

$$\xi_s(0) = \bar{\xi}_s,$$

and evaluate

$$\Psi(\bar{\xi}_0, \ldots, \bar{\xi}_n; \bar{t}) = [\xi_0(\bar{t})]^{M_0}.$$

In order to calculate the probability of treatment success, which is the same as the probability of extinction of the colony, we need to find the function value:

$$\lim_{t\to\infty} \varphi_{0,\ldots,0}(t) = \lim_{t\to\infty} \Psi(0, \ldots, 0; t).$$

The probability of treatment success. We assume that treatment starts at time t_*. The time t_* is assumed to correlate with the size N given by $N = \sum_s x_s(t_*)$, where the values x_s are the expected numbers of cells of each type. These are found from the following ODEs for the mean values:

$$\dot{x}_s = \sum_j l_j u^{in}_{s\to j} x^{in}_j + \left(l_s - d_s - \sum_i u^{out}_{s\to i} \right) x_s, \quad x_s(0) = \begin{cases} M_0, & s = 0, \\ 0, & \text{otherwise.} \end{cases}$$

In this paper we make the assumption that all the division and death rates in the absence of treatment are equal to each other. This allows us to solve the

above equations very easily to obtain the following simple connection between the time and the colony size: $N = M_0 exp\{(l-d)t_*\}$.

In order to calculate the probability of treatment success, we need to solve the equations for characteristics twice. The time axes is divided into two regions: $0 < t < t_*$ is pre-treatment, then $t > t_*$ is the treatment stage. First, we solve the equations for the treatment stage, that is, we find the limit $\lim_{t\to\infty} \xi_s(t)$. Then we use these values as initial conditions and solve the system for the pre-treatment phase, for the duration $t = t_*$. The resulting value of $\xi_0(t_*)$ is used to calculate

$$\lim_{t\to\infty} \Psi(0,\ldots,0;t) = (\xi_0(t_*))^{M_0}.$$

The systems for characteristics are solved numerically by using a standard Runge-Kutta ODE solver. The probability of treatment success given that the colony has grown to size N, is obtained as follows:

$$P(treat.\ succ.) = \frac{(\xi_0(t_*))^{M_0} - (d/l)^{M_0}}{1-(d/l)^{M_0}}.$$

This is because the probability of extinction ($(\xi_0(t_*))^{M_0}$) equals probability of going extinct before reaching size N ($(d/l)^{M_0}$) plus the probability to grow to size N and to be treated ($P(treat.\ succ.)$).

Symmetry assumptions. In our calculation we will use several symmetry assumptions, which allow for a very concise representation of results, but are not necessary in principle. As mentioned above, we assume that all the division and natural death rates are equal to each other. The other assumption concerns the death rates of cells. Let us suppose that we apply treatment with m drugs. In principle, the more drugs the cell is susceptible to, the higher is its death rate. However in this paper we will assume that if a cell is susceptible to at least one of the m drugs, it dies with the same rate as a fully susceptible cell. This death rate is equal to the natural death rate of untreated cells, d, plus a drug-related factor, h. On the other hand, if a cell is resistant to all the drugs, then its death rate is equal to that of the untreated cells, d.

Modeling cross-resistant mutations. The existing medical knowledge on the types of mutations for CML drugs is incorporated in the rates of mutations u. Here we illustrate it with the example of the drugs Imatinib, Dasatinib and Nilotinib. We assume that k_i mutations give rise to resistance to drug i without affecting resistance properties with respect to the other drugs (the index i takes values $1, 2, 3$ for the three drugs). This translates into $u_i = k_i\mu$, where μ is the basic point mutation rate. In our simulations we took k_i to be the same for all three drugs, $k_i = k$. This is another symmetry assumption which simplifies the system but is not necessary in principle. For the lack of exact measurements of this parameter, we experimented with different values of k_i in a biologically plausible range.

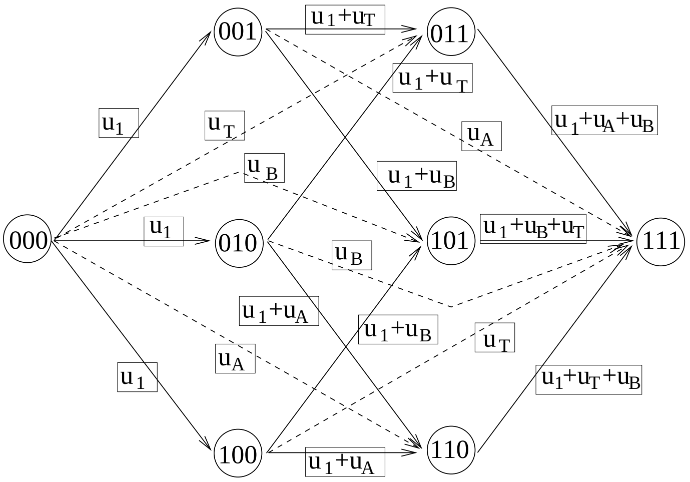

Figure 2: Combinatorial mutation diagram for a three-drug treatment with pairwise cross-resistant mutations. A mutation event (at rate u_T) is assumed to give rise to resistance against drugs 2 and 3; another mutation event at rate u_A - against drugs 1 and 2, and a third one (rate u_B) - against drugs 1 and 3. All one-drug resistant mutations are assumed to happen at rate u_1. The mutation rates are marked in rectangles for each edge of the diagram.

Further we include the cross-resistant mutation T315 by setting $u_{000\to111} = \mu$. There are no known mutations which confer resistance to only two of the three drugs; therefore, we set the rate of mutation to be zero for the following processes: $000 \to 011, 000 \to 101, 000 \to 110$. Note that the T315 mutation contributes to the following processes: $001 \to 111, 010 \to 111, 100 \to 111$ (the corresponding mutation rates are set to be equal μ), and $011 \to 111, 101 \to 111, 110 \to 111$ (the corresponding mutation rates are set to be equal $(k+1)\mu$).

We have also investigated the role of two-drug cross-resistant mutations. We assumed that there is a mutation event which gives rise to resistance against drugs 2 and 3; another mutation event - against drugs 1 and 2, and a third one - against drugs 1 and 3. The corresponding mutation rates are denoted as u_T, u_B and u_A, see diagram of figure 2. For simplicity we assume that all one-drug resistant mutations happen at the same rate, u_1. In general, there are five possible three-drug cross-resistance networks, see figure 3. In the figure, each drug is represented as a node of the network, and a connection corresponds to the existence of a mutation which confers cross-resistance to both drugs. Different types of line (dashed, solid, double) correspond to different mutants. If the lines are the same then triple cross-resistance takes place. In figure 3 we provide examples of the drugs which participate in each cross-resistance network. There, "I" stands for Imatinib, "D" for Dasatinib and "N" for Nilotinib. "K" stands for one of the drugs still in development which is active against cells with a T315I mutation, see main text. By setting some of the cross-resistance rates to zero, we can model four of the five possible cross-resistance networks

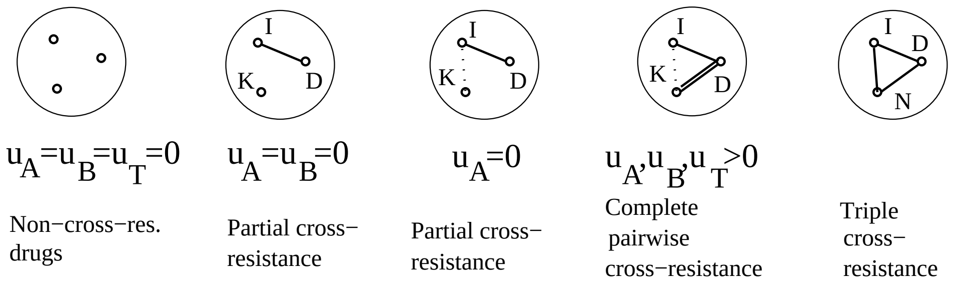

Figure 3: All possible three-drug resistance networks. The first four cases can be modeled by the framework of figure 2 and comprise all possible pairwise cross-resistance possibilities. The fifth network requires a triple-cross resistant mutant, figure 1.

by using the system presented in figure 2, from no cross-resistance to complete pairwise cross-resistance. The triple cross-resistance network was handled with equations corresponding to figure 1.

Back-mutations. No back mutations are included in the above equations. We have performed a separate study where all possible back-mutations were taken into account. This results in additional terms in the equations for characteristics. However, for biologically reasonable values of the mutation rates, the inclusion of back mutation did not make a measurable difference in the probability of treatment success.

A note on the statistical significance of our results. The stochastic model we use in this paper is a very convenient tool to convey comparative studies of different scenarios and treatment strategies. In this context, our method has significant advantage compared to, say, directly running stochastic simulations. If we were running stochastic simulations, and obtained two different results for two treatment strategies, then the question would be if the difference is "real" (or significant), or if it is only a result of stochastic fluctuations. This question does not arise with our method. Instead of running stochastic simulations, we operate directly with probabilities. For example, in figure 1 of the main paper we plot the probability of treatment success as a function of the tumor load. The difference in this quantity for the two closest curves in that figure, say for $\log_{10} N = 13.5$, is about 0.15, which is a 15% increase in the probability of treatment success (here, we compare three-drug combinations with two and three pairs of cross-resistant drugs). There is no stochastic noise here to make the difference an artifact of fluctuations, so we can interpret it directly. Therefore, the issue of "significance" in this context is not a mathematical or statistical question anymore, but rather a moral and financial issue, to be decided in accordance with some guidelines.

References

[1] Komarova, N.L. & Wodarz, D. (2005) Drug resistance in cancer: principles of emergence and prevention. Proc. Natl. Acad. Sci. 102 (27), pp. 9714-9.

[2] Komarova, N.L. (2006) Stochastic modeling of drug resistance in cancer. Jour. Theor. Biol. 239(3), pp. 351-366.

[3] Komarova, N.L. & Wodarz, D. (2007) Effect of cellular quiescence on the success of targeted CML therapy. PLoS ONE 2(10):e990.

[4] Komarova, N.L. & Wodarz, D. (2007) Stochastic modeling of cellular colonies with quiescence: an application to drug resistance in cancer. Theor Popul Biol. 72(4), pp. 523-38.
